# Sudden Appearance of Indurated Erythematous Plaques on a Man's Face

**DOI:** 10.1155/2016/5192689

**Published:** 2016-09-15

**Authors:** A. Carter, K. Viswanathan, K. Shulman

**Affiliations:** ^1^New York Medical College, New York, NY 10029, USA; ^2^Woodhull Medical Center, Brooklyn, NY 11206, USA

## Abstract

Rosacea fulminans (RF), previously known as pyoderma faciale, is a rare presentation of rosacea mostly seen in young women. RF is seen very rarely in men. We present below a case of a fifty-year-old male who presented with RF and was successfully treated with a combination of corticosteroids and isotretinoin.

## 1. Introduction

Rosacea fulminans was first reported in 1940 by O'Liery and Kierland of the Mayo Clinic in Rochester, Minnesota. We present below a rare presentation of RF in a man, his clinical course, and response to treatment.

## 2. Case Report

A fifty-year-old man presented with the sudden onset of a diffuse facial rash. He had a several years' history of rosacea, well controlled with only topical treatments with the last outbreak approximately two years prior to his presentation. His medical history included diabetes mellitus for which he took metformin. A review of system was positive only for dryness in the left eye.

**Figure 1:**
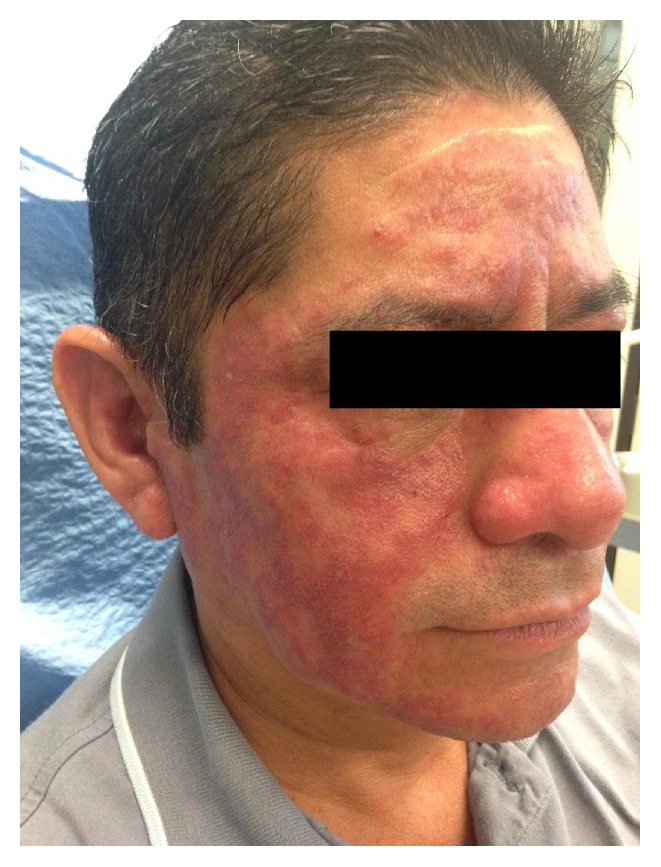
Initial presentation demonstrating indurated, erythematous plaques covering most of his forehead, cheeks, nose, and chin.

**Figure 2:**
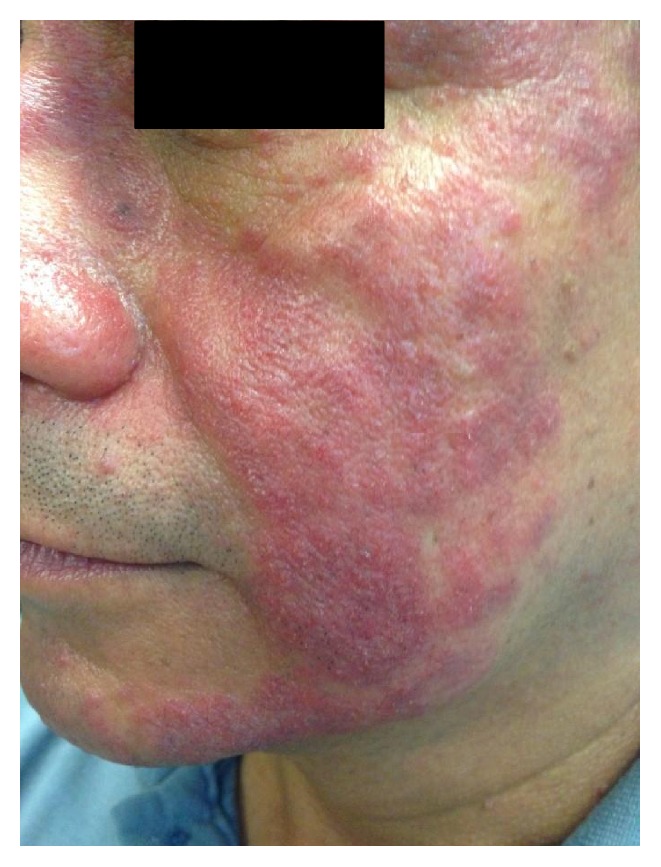
Profile view demonstrating erythematous, indurated papules and plaques as well as pustules with a sharp demarcation of the erythema to surrounding normal skin.

Physical exam revealed indurated, erythematous plaques covering most of his forehead, bilateral cheeks, including the nasolabial folds, nose, and chin. Numerous pustules and erythematous papules were also present. No cysts were noted on exam; and his back, chest, arms, and neck were spared. The affected areas were sharply demarcated from the unaffected areas (Figures 1 and 2). Mild erythema was seen in the medial sclera of his left eye.

His CBC and CMP were normal and his ANA titer was negative. A culture of his pustule returned no growth.

**Figure 3:**
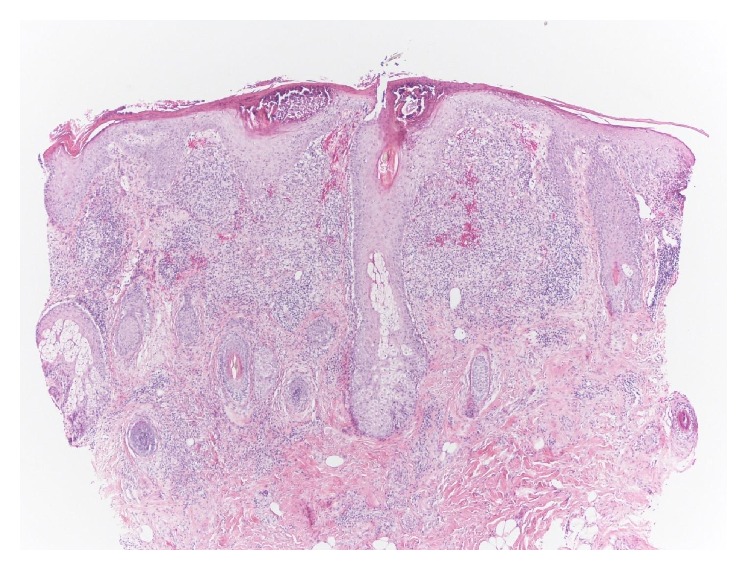
Hematoxylin-eosin biopsy specimen showing dense folliculocentric lymphohistiocytic infiltrate with a pustule and a demodex mite (40x).

**Figure 4:**
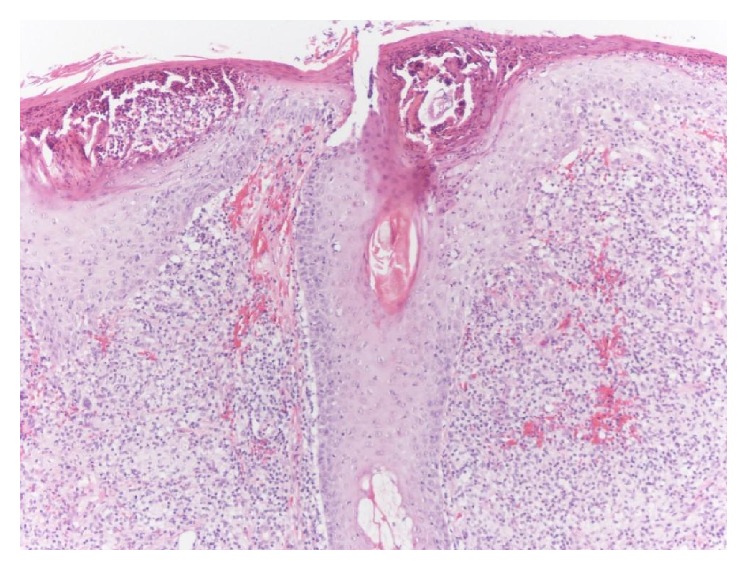
Further magnification highlights the folliculocentric inflammation (100x).

Hematoxylin-eosin staining showed granulomatous perifolliculitis and spongiosis with crusting. There was a heavy folliculocentric infiltrate of lymphocytes and histiocytes in the upper to mid dermis (Figures 3 and 4). This abrupt presentation, history of rosacea, physical exam findings, absence of any growth in his pustules, and the histopathology supported a diagnosis of rosacea fulminans (RF).

He was started on prednisone 40 mg daily along with risedronate 35 mg once a week, omeprazole 20 mg once a day, and calcium and vitamin D supplements. Isotretinoin 20 mg per day was added to his regimen three weeks after starting prednisone. Once on isotretinoin, prednisone was tapered down by 5 mg every week. When prednisone was tapered to 30 mg daily, isotretinoin was increased to 40 mg daily. Prednisone was then tapered over the next three weeks. We treated the patient to a goal of 150 mg/kg of isotretinoin over a five-month period. He had an excellent clinical response: clearance was seen by month two of the initiation of isotretinoin treatment. Three months after treatment completion, the patient required only topical metronidazole cream and sunscreen for maintenance of his rosacea.

## 3. Discussion

Kligman et al., in 1992, proposed renaming the disease, until then called pyoderma faciale, to rosacea fulminans since the progression of disease closely resembles acne fulminans; and similar to acne fulminans it presents with papules, pustules, and nodules [1]. It can be easily distinguished from acne vulgaris by the rapidity of its appearance and its fulminant course and by the rarity of comedones [2]. A patient with RF also tends to give a history of oily skin or seborrhea, which may help with the initial diagnosis [1, 2]. The majority of RF presentations are seen in women and very rarely in men [3]. While it predominantly affects the face, it can rarely affect regions just below the face [1].

Histologically, the presentation includes an extensive perifollicular inflammatory infiltrate, composed of a mixture of lymphocytes, histiocytes, and polymorphonuclear leukocytes. Necrosis of the connective tissue with extension to the surrounding tissues and into the pilosebaceous units, loss of collagen bundles and elastic tissue, and foreign body giant cell reaction have also been reported [4].

One mechanism proposed for the development of RF includes cytokine-mediated inflammation triggered by sebocytes [5]. However, outbreaks have been reported in a variety of pathophysiological settings: after ingestion of high dose vitamin B supplements [6]; with Crohn's disease and ulcerative colitis [7]; and during pregnancy [8].

The suggested treatment course includes systemic corticosteroids followed by isotretinoin. Topical corticosteroids can be useful adjuvants. Amongst all presentations of rosacea, RF is the only clinical setting when oral or topical corticosteroids are recommended [2]. Isotretinoin, in addition to its anti-inflammatory effects, is effective in RF through its antiseborrheic effects [9].

As an alternative to isotretinoin either macrolides or tetracyclines have effect through their anti-inflammatory properties [10]. Subantimicrobial dosing of doxycycline has also been shown to be effective in treating RF with extrafacial lesions [3]. During pregnancy, RF has been demonstrated to respond well to treatment with azithromycin [8]. In instances where the combination of corticosteroids and isotretinoin was ineffective, dapsone, through its anti-inflammatory properties, has been reported to be effective [5].

The combination of corticosteroids and isotretinoin remains the first-line treatment modality for RF [10]. It was highly effective in our patient and no recurrence has been reported as of submission of this case report.

## References

[1] Plewig G., Jansen T., Kligman A. M. (1992). Pyoderma faciale: a review and report of 20 additional cases: Is it rosacea?. *Archives of Dermatology*.

[2] Jansen T., Plewig G., Kligman A. M. (1994). Diagnosis and treatment of rosacea fulminans. *Dermatology*.

[3] Smith L. A., Meehan S. A., Cohen D. E. (2014). Rosacea fulminans with extrafacial lesions in an elderly man: successful treatment with subantimicrobial-dose doxycycline. *Journal of Drugs in Dermatology*.

[4] Massa M. C., Daniel Su W. P. (1982). Pyoderma faciale: a clinical study of twenty-nine patients. *Journal of the American Academy of Dermatology*.

[5] Bormann G., Gaber G., Fischer M., Marsch W. C. (2001). Dapsone in rosacea fulminans. *Journal of the European Academy of Dermatology and Venereology*.

[6] Jansen T., Romiti R., Kreuter A., Altmeyer P. (2001). Rosacea fulminans triggered by high-dose vitamins B6 and B12. *Journal of the European Academy of Dermatology and Venereology*.

[7] Razeghi S., Halvorson C. R., Gaspari A. A., Cross R. K. (2013). Successful treatment of localized pyoderma faciale in a patient with Crohn's disease. *Gastroenterology and Hepatology*.

[8] Fuentelsaz V., Ara M., Corredera C., Lezcano V., Juberias P., Carapeto F. J. (2011). Rosacea fulminans in pregnancy: successful treatment with azithromycin. *Clinical and Experimental Dermatology*.

[9] Thielitz A., Gollnick H. (2011). Rosacea. *Hautarzt*.

[10] D'Erme A. M., Boca A., Sabau M., Milanesi N., Simonacci F., Gola M. (2016). Successful treatment of rosacea fulminans in a 59-year-old woman with macrolide antibiotics and prednisone. *International Journal of Dermatology*.

